# Accuracy of mucocutaneous leishmaniasis diagnosis using polymerase chain reaction: systematic literature review and meta-analysis

**DOI:** 10.1590/0074-02760140280

**Published:** 2015-04

**Authors:** Ciro Martins Gomes, Suleimy Cristina Mazin, Elisa Raphael dos Santos, Mariana Vicente Cesetti, Guilherme Albergaria Brízida Bächtold, João Henrique de Freitas Cordeiro, Fabrício Claudino Estrela Terra Theodoro, Fabiana dos Santos Damasco, Sebastián Andrés Vernal Carranza, Adriana de Oliveira Santos, Ana Maria Roselino, Raimunda Nonata Ribeiro Sampaio

**Affiliations:** 1 Programa de Pós-Graduação em Clínica Médica; 2 Departamento de Neurociências e Ciências do Comportamento; 7 Laboratório de Biologia Molecular, Divisão de Dermatologia, Departamento de Clínica Médica, Hospital das Clínicas, Faculdade de Medicina de Ribeirão Preto, Universidade de São Paulo, Ribeirão Preto, SP, Brasil; 3 Serviço de Dermatologia; 4 Departamento de Clínica Médica; 5 Programa de Pós-Graduação em Ciências Médicas, Faculdade de Medicina, Universidade de Brasília, Brasília, DF, Brasil; 6 Serviço de Dermatologia, Hospital Regional da Asa Norte, Brasília, DF, Brasil

**Keywords:** leishmaniasis, mucocutaneous, diagnosis, polymerase chain reaction

## Abstract

The diagnosis of mucocutaneous leishmaniasis (MCL) is hampered by the absence of a gold standard. An accurate diagnosis is essential because of the high toxicity of the medications for the disease. This study aimed to assess the ability of polymerase chain reaction (PCR) to identify MCL and to compare these results with clinical research recently published by the authors. A systematic literature review based on the Preferred Reporting Items for Systematic Reviews and Meta-Analyses: the PRISMA Statement was performed using comprehensive search criteria and communication with the authors. A meta-analysis considering the estimates of the univariate and bivariate models was performed. Specificity near 100% was common among the papers. The primary reason for accuracy differences was sensitivity. The meta-analysis, which was only possible for PCR samples of lesion fragments, revealed a sensitivity of 71% [95% confidence interval (CI) = 0.59; 0.81] and a specificity of 93% (95% CI = 0.83; 0.98) in the bivariate model. The search for measures that could increase the sensitivity of PCR should be encouraged. The quality of the collected material and the optimisation of the amplification of genetic material should be prioritised.

American tegumentary leishmaniasis (ATL) is caused by parasites of the *Leishmania* genre. The clinical manifestations of ATL are predominantly classified as cutaneous (CL) or mucocutaneous leishmaniasis (MCL), depending on the absence of mucous lesions or on the involvement of mucous membranes, respectively (Gomes et al. 2014 b).

The etiologic diagnosis of ATL in all its forms is a difficult task. This difficulty is increased in cases of MCL, in which sampling frequently requires invasive techniques because of the potential presence of deep nasopharyngeal lesions (Maretti-Mira et al. 2011, Weinkopff et al. 2013).

The absence of a gold standard diagnostic test for MCL is a major obstacle. The existing tests cannot be independently used for the diagnosis of MCL because the accuracy of the tests is unsatisfactory. The classical diagnostic methods for MCL include immunological and parasitological tests. The immunological tests include the intradermal leishmanin test (the Montenegro skin test) and serological tests, which are known for their high sensitivity; however, these immunological tests exhibit low diagnostic specificity (Ferreira et al. 2006, Gomes et al. 2014a). The parasitological tests aim to directly identify the parasite and primarily consist of direct tests, culturing and histopathological examinations. Contrary to the initial tests, these latter tests have good specificity; however, they also exhibit poor sensitivity (Gomes et al. 2014a).

Studies have indicated that molecular biology techniques, particularly polymerase chain reaction (PCR), offer good sensitivity and specificity and, ultimately, increased accuracy compared with immunological and parasitological tests (Medeiros et al. 2002, Garcia et al. 2005, Silva et al. 2012). However, several methodologies are used, making it difficult to generate simple comparisons among the papers.

We performed a systematic literature review of studies of the diagnostic accuracy of PCR in detecting MCL as part of our groups' effort to develop non-invasive collection methods for the diagnosis of MCL. The primary objective of this study was to assess the status of various molecular biology techniques in recognising MCL and to compare these data with the recent clinical research published by our group (Gomes et al. 2014a).

## MATERIALS AND METHODS

This systematic literature review was performed based on recommendations by the Preferred Reporting Items for Systematic Reviews and Meta-Analyses: the PRISMA Statement, with adjustments for evaluating the diagnostic accuracy of the studies (Devillé et al. 2002, Leeflang et al. 2008 , Moher et al. 2009, Leeflang 2014). This review is registered with the platform PROSPERO - International Prospective Register of Systematic Review (University of York, UK), with the identifier CRD42014007038.

*Paper selection* - In January 2013, the following search terms were generated: "((((((mucosal) OR mucocutaneous) OR mucous)) AND leishmaniasis) AND diagnosis) AND Polymerase Chain Reaction", using the advanced search system on the PubMed site (PubMed Advanced Search Builder) following the recommendations for searches with comprehensive criteria. Because MCL is a highly endemic disease in South America, predominantly in Brazil, we included the non-MeSH terms "mucous" and "mucosal", which are frequently translated from the Portuguese words *leishmaniose mucosa.*

Two researchers were recruited to search the related references in the following databases or virtual libraries: PubMed, EMBASE, Web of Knowledge, SciELO and LILACS, using the same terms and connectors through each specific advanced search tool (Table I). No specific date was defined for applying the search criteria by each examiner. The paper search began in January 2013 and was concluded in December 2013. The inclusion and exclusion criteria for the paper are detailed below.

**TABLE I:** URLs for the search sources used in the systematic literature review that were accessed to conduct the survey of related articles

Database or virtual library	Link
PubMed	ncbi.nlm.nih.gov/pubmed/
EMBASE	aplicacao.periodicos.saude.gov.br/autenticar/profissional
Web of Knowledge	apps.webofknowledge.com/UA_GeneralSearch_input.do?highlighted_tab=UA&product=UA&search_mode=GeneralSearch&SID=4Dlh@39Jdd364KmNbFe&last_prod=WOS&highlighted_tab=UA&cacheurl=no
SciELO	scielo.org/php/index.php
LILACS	lilacs.bvsalud.org/

*Inclusion criteria - *Papers that fulfilled all of the following criteria were selected and included. Addressed the topic: using PCR techniques for the diagnosis of MCL leishmaniasis, studies in humans, cases of mucosal leishmaniasis potentially associated with skin lesions, more than seven cases of mucosal leishmaniasis, any number of controls without a diagnosis of mucosal leishmaniasis, no restriction for defining the controls (healthy, with mucosal or skin lesions) and written in English, French, Portuguese or Spanish.

*Exclusion criteria - *Fewer than eight cases of mucosal leishmaniasis, studies without controls, studies that used known cured cases of ATL as controls, studies in which patients with CL and mucosal leishmaniasis were included without being explicitly separated, PCR tests conducted with cultured patient samples, only the presence of exclusively CL forms of ATL and written in languages other than English, French, Portuguese or Spanish.

No restrictions were imposed on the paper selection process regarding the year of publication and no additional limits were set. Additionally, no restrictions were imposed regarding the reference composite standard for the diagnostic criteria of MCL. This characteristic was considered for the classification of selected papers and for the inclusion in the final meta-analysis model.

The papers were accessed using the periodicals portal available through the Brazilian Federal Agency for Support and Evaluation of Graduate Education on the internet-connected network of the University of Brasília and through direct contact with the authors *via *electronic correspondence.

After the search and review by the researchers, any disagreements were resolved by a third evaluator, who was blind to the identity of the researchers who conducted the previous selection. Electronic communications were sent to all of the corresponding authors of the selected papers with an inquiry regarding whether they knew of any published or unpublished study that addressed the criteria of interest. Additionally, the references cited in the pre-selected papers were examined for additional relevant studies. Thesis databases at several internationally recognised universities were searched as well (Supplementary data I).

The researchers constructed a 2 x 2 table based on the information contained in the papers with the aim of calculating the sensitivity, specificity, predictive values and accuracy using OpenEpi^(r)^ v.3.01 (Emory University, Rollins School of Public Health, USA). After this procedure, the corresponding authors of the selected papers were again contacted by email with the intent of retrieving the data of papers in which it was impossible to construct the 2 x 2 table and to confirm the precision of the accuracy data obtained after reading the papers.

*Analysis of the selected papers* - Two reviewers conducted the qualitative analysis and paper critiques. The qualitative assessment was based on the completion of the tables, which were constructed during the above-mentioned clinical study previously published by this group (Table II) (Gomes et al. 2014a). Any discrepancies in completing the qualitative table were resolved using an evaluation by a third examiner.

**TABLE II:** A qualitative analysis tool created by the author based on clinical research previously conducted by this group^a^

Was there a sample size calculation?
Study site
Date of the study
Number of cases/positivity of the test performed
Inclusion criteria for the cases
Number of controls/positivity of the test performed
Inclusion criteria for the controls
Sample studied
Method of DNA extraction
Primers used to amplify the genetic material, location of the target gene sequence and molecular weight of the amplified material
Method for visualising the amplified material
Sensitivity, specificity, positive predictive value, negative predictive value, accuracy and odds ratio of test evaluated
Species identified

^a:^ (Gomes et al. 2014a).

The critical analysis was completed using the "QUADAS tool: a tool for the quality assessment of studies of diagnostic accuracy included in systematic reviews*"* (Whiting et al. 2006), which consists of 14 items rated as "yes", "no" or "unclear". Discrepancies between the data entered by the two evaluators were recorded under the ''unclear'' classification.

The decision regarding which results would be included in the meta-analysis was primarily resolved using criteria related to the test methodology. In this step, the study previously published by our group, which was the basis for performing this systematic review, was included (Gomes et al. 2014a).

*Analysis of the heterogeneity of the papers* - In the first step, the papers were separated according to the composite reference standard used to define the cases and controls. The papers were classified according to the use of clinical, immunological, or/and parasitological criteria (Table III).

**TABLE III:** Characteristics of the studies selected

Reference	Location	Design	Cases included	Sample	Size of the amplicons (bp)
Piñero et al. (1999)	Cuzco/Peru	Case-control	Parasitological	Blood	72
Pirmez et al. (1999)	Rio de Janeiro/Brazil	Cross-sectional/cohort	Clinical	Biopsy	120
Victoir et al. (2003)	Lima/Peru	Cross-sectional/cohort	Polymerase chain reaction (PCR)	Biopsy	870
Disch et al. (2005)	Belo Horizonte/Brazil	Case-control	Clinical, immunological and parasitological	Biopsy	51,120
Camera et al. (2006)	Rio de Janeiro/Brazil	Case-control	Parasitological and PCR	Blood	120
Bracho et al. (2007)	Bogotá/Colombia	Case-control	Clinical, immunological and parasitological	Biopsy	120, 1,000
Deborggraev et al. (2008)	Lima/Peru	Case-control	Parasitological	Aspirate	115
Pereira et al. (2008)	Paraná/Brazil	Cross-sectional/cohort	Parasitological and PCR	Biopsy	70, 750, 103, 62
Fagundes et al. (2010)	Rio de Janeiro/Brazil	Cross-sectional/cohort	Parasitological	Biopsy	120
Boggild et al. (2011)	Lima/Peru	Case-control	Clinical, immunological, parasitological and PCR	Swab, nasal brush and biopsy	70
Thomaz-Soccol et al. (2011)	Paraná/Brazil	Cross-sectional/cohort	Clinical, parasitological and PCR	Biopsy	70, 750
Veland et al. (2011)	Lima/Peru	Case-control	Parasitological and PCR	Urine	70
Marco et al. (2012)	Salta/Argentina	Cross-sectional/cohort	Clinical, immunological and parasitological	Lesion scraping	168, 700, 103, 79, 79, 862
Neitzke-Abreu et al. (2013)	Maringá/Brazil	Cross-sectional/ cohort	Clinical, immunological and parasitological	Blood	70

In the second step, the following characteristics were considered: the sample analysed, molecular weight and the PCR primer target. Human tissue samples were considered similar whether they were stored in filter paper, paraffinised or frozen (Marques et al. 2001). The DNA extraction methods that used commercial kits or phenol-extraction techniques were considered similar (Marques et al. 2001). Additionally, the studies that used primers that targeted the kDNA mini-circle of *Leishmania* spp and whose amplicons were less than 150 bp were considered similar. Statistical tests to evaluate the heterogeneity among the selected studies were not performed because of the limited number of papers with comparable methodologies.

*Creating pooled forest plots* - A sensitivity analysis considering the estimates of the univariate and bivariate models was performed (Deppen et al. 2014). The univariate model was estimated with Meta-DiSc^(r)^ software (Ramón y Cajal University Hospital, Spain) and the bivariate model was fitted in R^(r)^ software, v.3.1.2, using the mada package (Institute for Statistics and Mathematics of Wirtschaftsuniversität, Austria). This library estimates the parameters by the restricted maximum likelihood method. All of the tests were bimodal (2-sided) and applied a 5% type I error rate.

## RESULTS

**Fig. 1:**
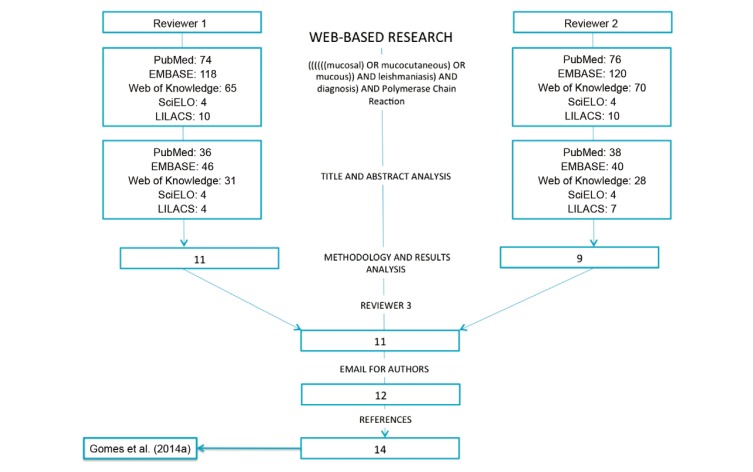
a flowchart of paper selection and inclusion.

At the end of the selection process, 14 papers were included based on the above-mentioned criteria (Fig. 1, Supplementary data II): Piñero et al. (1999), Pirmez et al. (1999), Victoir et al. (2003), Disch et al. (2005), Camera et al. (2006), Bracho et al. (2007), Deborggraeve et al. (2008), Pereira et al. (2008), Fagundes et al. (2010), Boggild et al. (2011), Thomaz-Soccol et al. (2011), Veland et al. (2011), Marco et al. (2012) and Neitzke-Abreu et al. (2013).

*Qualitative meta-synthesis of the results* - Regarding location, seven studies were conducted in Brazil, all of which were in the south and southeastern regions of the country, in which *Leishmania (Viannia) braziliensis* species are endemic. Five studies were conducted in Peru, one in Colombia and one in Argentina. Five of the 14 papers included only patients with MCL. The remaining studies combined cases of MCL and CL.

One of the main reasons for heterogeneity among the selected papers was the composite reference standard used to define cases of MCL and controls. The patients were selected based on the compilation of clinical, epidemiological and parasitological criteria in only three studies. A common error observed in six papers was the inclusion of a PCR test to determine the inclusion and allocation criteria. Of the remaining studies, three used parasitological criteria, one used clinical criteria and one did not report the diagnostic criteria used.

One paper clearly stated the calculation of a sample size (Boggild et al. 2011) considering only the expected sensitivity of the tested diagnostic methods. Seven papers used a case-control design, whereas the other one-half of the papers used a cross-sectional/cohort design for the recruitment and allocation (Table III).

With respect to the type of samples analysed, three used blood samples, one used urine samples, one used lesion scrapings, one used lesion aspirates, one used nasal swab*s* and biopsies and seven used tissue samples collected by biopsy. The data on the positivity and characteristics of the tests are detailed in Tables II, IV. The studies that exhibited greater accuracy for MCL using PCR were performed using tissue samples (Deborggraeve et al. 2008, Boggild et al. 2011).

**TABLE IV:** Test properties calculated after creating a 2 x 2 table

Reference	Cases^*a*^	Controls^*b*^	Sensitivity (95% CI)	Specificity (95% CI)	PPV (95% CI)	NPV (95% CI)	Accuracy (95% CI)
Piñero et al. (1999)	36 (30)	19 (0)	83.33 (68.11-92.13)	100 (83.18-100)	100 (88.65-100)	76.00 (56.57-88.5)	89.09 (78.17-94.9)
Pirmez et al. (1999)	30 (23)	14 (0)	76.67 (59.07-88.21)	100 (78.47-100)	100 (85.69-100)	66.67 (45.37-82.81)	84.09 (70.63-92.07)
Victoir et al. (2003)	15 (11)	5 (0)	73.33 (48.05-89.1)	100 (56.55-100)	100 (74.12-100)	55.56 (26.66-81.12)	80 (58.4-91.03)
Disch et al. (2005) 51 ^*c*^	13 (11)	10 (0)	84.62 (57.76-95.67)	100 (75.25-100)	100 (74.12-100)	83.33 (55.2-95.3)	91.3 (73.2-97.58)
Disch et al. (2005) 120 ^*c*^	13 (11)	10 (0)	84.62 (57.76-95.67)	100 (75.25-100)	100 (74.12-100)	83.33 (55.2-95.3)	91.3 (73.2-97.58)
Camera et al. (2006)	20 (7)	37 (9)	35 (18.12-56.71)	75.68 (59.88-86.64)	43.75 (23.1-66.82)	68.29 (53.02-80.44)	61.4 (48.43-72.94)
Bracho et al. (2007) kDNA ^*d*^	35 (24)	25 (2)	68.57 (52.02-81.45)	92 (75.03-97.78)	92.31 (75.86-97.86)	67.65 (50.84-80.87)	78.33 (66.38-86.88)
Bracho et al. (2007) ITS ^*d*^	35 (14)	25 (1)	40 (25.55-56.43)	96 (80.46-99.29)	93.33 (70.18-98.81)	53.33 (39.08-67.07)	63.33 (50.68-74.38)
Deborggraeve et al. (2008)	12 (11)	8 (0)	91.67 (64.61-98.51)	100 (67.56-100)	100 (74.12-100)	88.89 (56.5-98.01)	95 (76.39-99.11)
Pereira et al. (2008)	14 (12)	3 (0)	85.71 (60.06-95.99)	100 (43.85-100)	100 (75.15-100)	60 (23.07-88.24)	88.24 (65.66-96.71)
Fagundes et al. (2010)	15 (14)	15 (1)	93.33 (70.18-98.81)	93.33 (70.18-98.81)	93.33 (70.18-98.81)	93.33 (70.18-98.81)	93.33 (78.68-98.15)
Boggild et al. (2011) Cervisoft ^®^	23 (22)	10 (1)	95.65 (79.01-99.23)	90 (59.58-98.21)	95.65 (79.01-99.23)	90 (59.58-98.21)	93.94 (80.39-98.32)
Boggild et al. (2011) Hystobrush ^®^	23 (21)	10 (1)	91.3 (73.20-97.58)	90 (59.58-98.21)	95.45 (78.20-99.19)	81.82 (52.30-94.86)	90.91 (76.43-96.86)
Boggild et al. (2011) Biopsy ^*e*^	23 (22)	5 (0)	95.65 (70.01-99.23)	100 (56.55-100)	100 (85.13-100)	83.33 (43.65-96.99)	96.43 (82.29-99.37)
Thomaz-Soccol et al. (2011) MP1L/MP3H ^*f*^	15 (15)	6 (0)	100 (79.61-100)	100 (60.97-100)	100 (79.61-100)	100 (60.97-100)	100 (84.54-100)
Thomaz-Soccol et al. (2011) B1/B2 ^*f*^	15 (15)	6 (0)	100 (79.61-100)	100 (60.97-100)	100 (79.61-100)	100 (60.97-100)	100 (84.54-100)
Veland et al. (2011)	8 (6)	32 (0)	75 (43.93-92.85)	100 (89.28-100)	100 (60.97-100)	94.12 (80.91-98.37)	95 (83.5-98.62)
Marco et al. (2012)	9 (8)	19 (3)	88.89 (56.5-98.01)	84.21 (62.43-94.48)	72.73 (43.43-90.25)	94.12 (73.02-98.95)	85.71 (68.51-94.3)
Neitzke-Abreu et al. (2013) Blood ^*e *^	10 (6)	117 (2)	60 (31.27-83.18)	98.29 (93.98-99.53)	75 (40.93-92.85)	96.64 (91.68-98.69)	95.28 (90.08-97.82)
Gomes et al. (2014a) Biopsy ^*e*^	13 (8)	30 (0)	61.54 (35.52-82.29)	100 (88.65-100)	100 (67.56-100)	85.71 (70.62-93.74)	88.37 (75.52-94.93)

a: true positives; b: false positives; c: amplicon size; d: polymerase chain reaction target; e: collected material; f: primers; CI: confidence interval; NPV: negative predictive value; PPV: positive predictive value. Publications with more than one collection or amplification method were subdivided.

With respect to the properties of the diagnostic tests, specificity tended to be the highest. Ten papers had a specificity of 100% and two reported a specificity of less than 90%. The level of sensitivity reported in most of the papers was between 60-90%. Two papers reported sensitivities below 50% and one paper reported a sensitivity of 100%.

**Fig. 2:**
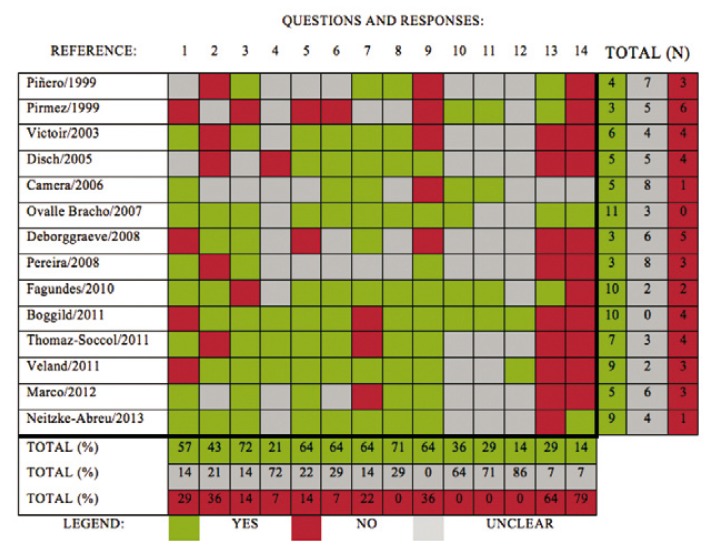
"QUADAS: a tool for the quality assessment of studies of diagnostic accuracy included in systematic reviews". The answers of the two examiners to the 14 items included in the tool. Disagreements between the two examiners were classified as "unclear".

*Critical analysis of the selected papers* - The analysis was performed using the QUADAS tool (Fig. 2) and indicated that the largest amount of data not reported in the papers (a "no" response) were related to the last two questions, which inquired about inconclusive results and records for the loss of patients in the studies. The cases in which the response was ''unclear'' were most frequently observed for questions 10, 11 and 12, thus indicating that the selected papers did not obviously report whether the examiner was blind to the reference standard during the application of the evaluated test and *vice versa*. This result additionally indicated that most of the papers did not explicitly detail whether the data evaluated in the present study were consistent with the data used in clinical practice.

Papers published during or after 2010 were of higher quality according to the QUADAS tool than the papers published prior to this date (Fig. 2).

*Creating pooled forest plots* - After the methodological evaluation, only three papers were considered sufficiently similar and were selected: Disch et al. (2005), Bracho et al. (2007) and Thomaz-Soccol et al. (2011). It was possible to compare the results in these studies to clinical research previously performed by this group; however, this comparison was restricted to tissue samples (Gomes et al. 2014a).

**Fig. 3:**
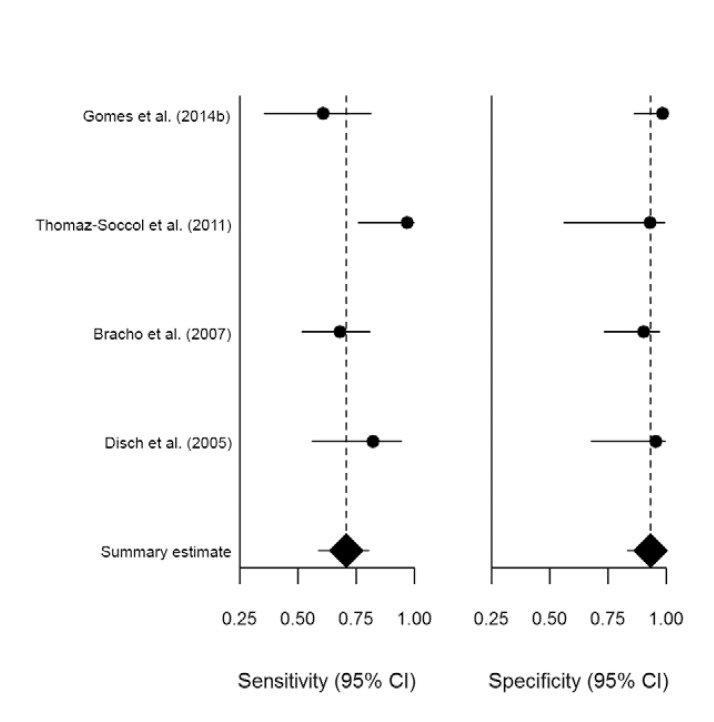
a forest plot of the pooled data for sensitivity and specificity - R(r) software v.3.1.2 using the mada package (Institute for Statistics and Mathematics of Wirtschaftsuniversität, Austria). CI: confidence interval.

**Fig. 4:**
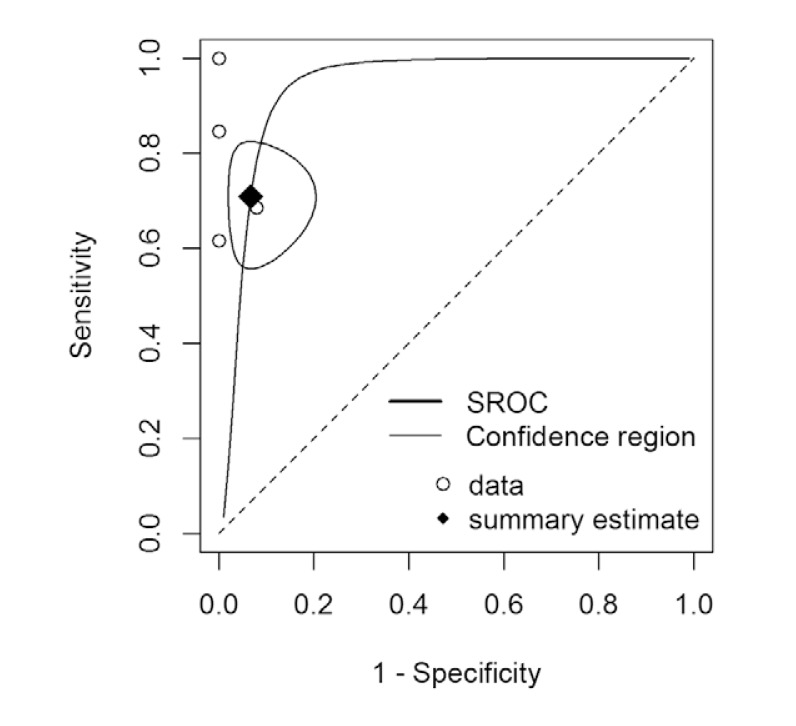
summary receiving operating characteristics curve (SROC): the relationship between sensitivity and specificity - R(r) software v.3.1.2 (Institute for Statistics and Mathematics of Wirtschaftsuniversität, Austria).

The joint estimates of sensitivity and specificity in the univariate and bivariate models are presented in Table V and Fig. 3. The area under the receiving operating characteristics (ROC) curve, considering the bivariate model, was estimated at 0.94 (Fig. 4).

**TABLE V:** Joint estimates of sensitivity and specificity in the univariate and bivariate models

	Univariate (95% CI)	Bivariate (95% CI)
Sensitivity	0.76 (0.65; 0.85)	0.71 (0.59; 0.81)
Specificity	0.97 (0.90; 1.00)	0.93 (0.83; 0.98)

CI: confidence interval.

## DISCUSSION

Some authors have reported that systematic reviews on diagnostic accuracy testing tend to result in studies of differing quality compared with reviews that examine clinical trials (Devillé et al. 2002, Leeflang et al. 2008, Leeflang 2014). Additionally, papers regarding diagnostic accuracy have considerable methodological heterogeneity compared with that of intervention studies. For this reason, it is not always possible to complete a meta-analysis (Devillé et al. 2002). The full methodological procedures are frequently not described within the papers and to understand these procedures, one must conduct a detailed reading of one or more papers cited by the authors.

*Definition of cases and controls (the use of a composite reference standard)* - The definition of cases and controls exerts a fundamental influence on the results of accuracy diagnostic studies. Variations in these criteria were one of the most frequent points of heterogeneity among the papers (Table III). Recent systematic reviews of the literature focused on the diagnosis of kala-azar have reported the same problems regarding the classification of cases and controls, which appears to be a major concern for all of the clinical manifestations of leishmaniasis, including MCL (Adams et al. 2013, de Ruiter et al. 2014).

Defining cases using only parasitological tests substantially increased the sensitivity of PCR because parasites were abundant in these cases. However, these case definitions did not correspond to clinical practice. Infection by* L. (V.) braziliensis* is known for its low density of parasites and severe local inflammation (Brelaz-de-Castro et al. 2012). This species is the primary cause of MCL and a considerable percentage of these patients do not exhibit positivity using parasitological techniques.

Additionally, defining controls is crucial. The possible inclusion of patients with previously cured MCL or those undergoing treatment might reduce the specificity of the tests. These patients might continue to harbour *Leishmania* cell debris or even the latent form of the parasite without manifesting the clinical disease (Mendonça et al. 2004). We evaluated the procedures used to define cases of MCL and controls (Tables II, III) to define which papers were comparable for inclusion in the meta-analysis model.

*Properties of the diagnostic tests* - The results revealed that the major factor responsible for the variations in diagnostic accuracy among the studies was the sensitivity value. It is difficult to assign a specific reason for increases or decreases in sensitivity. However, an unbiased analysis of the results supports the conclusion that specificity remains relatively stable even with extreme increases in sensitivity. Factors such as reductions in the molecular weight of the fragments amplified by primers and the use of commercial kits to extract DNA did not increase the proportion of false positive tests. This information was confirmed using an analysis of the generated the summary ROC curve (Fig. 4).

Random effects bivariate logit-normal sensitivity and specificity estimates are recommended for meta-analyses of diagnostic studies (Simel & Bossuyt 2009). However, these estimates are considered more complex methodologies (Simel & Bossuyt 2009). Some authors suggest that the use of univariate methods is not likely to result in significant changes. An analysis of the pooled results in the univariate and bivariate models confirmed similarities between the methods (Table V).

These results confirmed the high specificity of the PCR techniques for the diagnosis of MCL, which could be considered an important advantage compared with the unspecific immunological tests. In addition, the sensitivity was greater than the values described for the parasitological tests (Gomes et al. 2014b). These results confirm previous reports that consider PCR to be the most accurate method for the diagnosis of MCL (Gomes et al. 2014a).

*Limitations* - One of the most important limitations of this study is that we excluded studies with fewer than eight cases of MCL. This decision aimed to define a minimal relevance of studies because of an initial intention to include non-controlled studies and to use only simpler univariate methods of aggregation. This procedure was based on existing recommendations for sample size calculation in diagnostic test studies. As an example, a sample size of eight patients would only be sufficient if a low confidence interval inferior limit were set in association with an expected sensitivity level greater than 95%, which is not realistic for the diagnosis of MCL (Flahault et al. 2005, Bailly et al. 2014). We hypothesise that the existence of a considerable quantity of references with fewer than eight cases that fulfil all of the inclusion criteria is not probable. In addition, studies with a sample size that is insufficiently small would have a lower influence on the final result of the meta-analysis.

The use of QUADAS was selected instead of its more recent version QUADAS 2. Although the methods measure identical characteristics, the newer version allows a written description of four key domains (patient selection, index test, reference standard and flow/timing) (Whiting et al. 2011). Because we constructed a simplified tool that covered these domains and the domains related to molecular biology procedures (Table II), we decided to use the QUADAS for simplicity and to avoid the duplication of data collection. The absence of a third reviewer for the analysis of the QUADAS classification (Fig. 2) could have reduced the precision of this procedure. We reasonably considered that any mistake occurring during the classification would be highly influenced by an unclear description of the methodologies. In this case, the discrepancies were classified as "unclear".

The heterogeneous application of the composite reference standard in the selected papers ensures that it is difficult to separate the patients with CL or MCL. The simultaneous inclusion of these two forms of ATL was identified in nine studies, which may have increased the risk of bias during the extraction of the data from the studies. After constructing the 2 x 2 table and calculating the sensitivity, specificity, predictive values and accuracy, the authors of previously selected papers were contacted by email to retrieve the incomplete data and confirm the accuracy of the data. This approach aimed to reduce possible discrepancies between the accomplishments of the study and the possible interpretations of the scientific paper.

*Recommendations* - This systematic literature review analyses the available data on the PCR techniques used in the diagnosis of MCL. The significant methodological heterogeneity makes comparisons between studies more difficult.

Based on these results, it is necessary to generate a consensus and protocols that recommend optimised practices for PCR in MCL (da Graça et al. 2012). It is possible to infer that the use of techniques aimed to increase the sensitivity of the tests should be pursued, particularly because the specificity is generally satisfactory. The tissue samples collected directly from the lesion and the use of high-sensitivity extraction methods must be observed in the preparation for PCR processing. Additionally, the targeted DNA sequence must be observed because the primers that amplify the kDNA sequences of *Leishmania* exhibited better sensitivity than other sequences (van den Bogaart et al. 2013). Additionally, related techniques, such as real-time quantitative PCR, might be used to improve sensitivity (van den Bogaart et al. 2013).
